# Ternary Heterojunction Graphitic Carbon Nitride/Cupric Sulfide/Titanium Dioxide Photoelectrochemical Sensor for Sesamol Quantification and Antioxidant Synergism

**DOI:** 10.3390/bios13090859

**Published:** 2023-08-30

**Authors:** Likun Huang, Jingshi Yang, Zhishan Liang, Ruilian Liang, Hui Luo, Zhonghui Sun, Dongxue Han, Li Niu

**Affiliations:** 1 Guangzhou Key Laboratory of Sensing Materials & Devices, Center for Advanced Analytical Science, School of Chemistry and Chemical Engineering, Guangzhou University, Guangzhou 510006, China; 2112005015@e.gzhu.edu.cn (L.H.); 1905170041@e.gzhu.edu.cn (J.Y.); 1112116013@e.gzhu.edu.cn (Z.L.); 2005100039@e.gzhu.edu.cn (R.L.); 2005100011@e.gzhu.edu.cn (H.L.); cczhsun@gzhu.edu.cn (Z.S.); lniu@gzhu.edu.cn (L.N.); 2 Guangzhou Provincial Key Laboratory of Psychoactive Substance Monitoring and Safety, Anti-Drug Technology Center of Guangdong Province, Guangzhou 510230, China; 3 School of Chemical Engineering and Technology, Sun Yat-sen University, Zhuhai 519082, China

**Keywords:** photoelectrochemical sensor, sesamol, antioxidant synergism

## Abstract

Sesamol (SM) is a potent natural antioxidant that can quench free radicals and modulate the cholinergic system in the brain, thereby ameliorating memory and cognitive impairment in Alzheimer’s disease patients. Moreover, the total antioxidant capacity can be amplified by synergistic interactions between different antioxidants. Here, we constructed a ternary heterojunction graphitic carbon nitride/cupric sulfide/titanium dioxide (g-C_3_N_4_/CuS/TiO_2_) photoelectrochemical (PEC) sensor for the quantification of SM and its synergistic interactions with other antioxidants. Crucially, the Schottky barrier in ternary semiconductors considerably enhances electron transfer. The PEC sensor showed a wide linear range for SM detection, ranging from 2 to 1277 μmol L^−1^, and had a limit of detection of 1.8 μmol L^−1^. Remarkably, this sensing platform could evaluate the synergism between SM and five typical lipid-soluble antioxidants: *tert*-butyl hydroquinone, vitamin E, butyl hydroxyanisole, propyl gallate, and butylated hydroxytoluene. Owing to its low redox potential, SM could reduce antioxidant radicals and promote their regeneration, which increased the overall antioxidant performance. The g-C_3_N_4_/CuS/TiO_2_ PEC sensor exhibited high sensitivity, satisfactory selectivity, and stability, and was successfully applied for SM determination in both soybean and peanut oils. The findings of this study provide guidance for the development of nutritional foods, nutrition analysis, and the treatment of diseases caused by free radicals.

## 1. Introduction

Many people live unhealthy lifestyles, which often include smoking, drinking, getting insufficient sleep, and having an unbalanced diet, all of which result in the accumulation of a large number of oxidizing substances (reactive oxygen and nitrogen free radicals) in the body. These substances disrupt the redox balance of the body and damage biological macromolecules, organelles, and tissue [1]. This process is called oxidative stress [2]. Antioxidants are crucial for slowing down or preventing harmful oxidation and function by scavenging excess free radicals to combat oxidative stress and maintain redox homeostasis. Natural foods, such as vegetables, fruits, and tea, are rich in antioxidants, and their consumption can lower the risk of some chronic diseases [3]. Thus, there is interest in identifying natural sources of antioxidants and adding them to food formulations to improve human health and treat diseases [4]. Some natural antioxidants, such as vitamins E and C and natural polyphenols (catechins, resveratrol, and tea polyphenols), have received considerable attention from researchers because of their remarkable antioxidant ability and bioavailability [5]. Similarly, sesamol (SM) is a natural oil-soluble antioxidant extracted from sesame oil that has potent anti-aging, anti-mutagenic, and anti-cancer properties. SM is widely used in medicine and food technology [6,7]. For instance, sesame oil has shown potential in treating conditions such as cancer, neurodegenerative diseases, and acute liver injury. This is attributed to its ability to enhance the blood lipid profile and reduce oxidative stress. Moreover, SM has been found to influence the choline system, specifically by amplifying the activity of choline acetyltransferase and reducing that of cholinesterase. Such modulation benefits memory and cognitive functions, which are important in the prevention of Alzheimer’s disease (AD) [8]. However, few studies have focused on the determination of the antioxidant capacity of SM.

In view of the importance of SM in the fields of medicine and food technology [9], several detection and quantification methods, including electrochemical methods [10], thermogravimetric analysis [11], and spectrophotometry [12], have been developed. However, these technologies have disadvantages, such as low stability and reproducibility, time-consuming operating procedures, low detection limits, and susceptibility to interference, which hinder their large-scale application. Sensors have recently attracted considerable research attention owing to their high sensitivity, low cost, and rapid response [13]. Photoelectrochemistry (PEC) is an innovative technique that combines the advantages of photochemistry and electrochemistry [14]. However, to date, a PEC sensor for the detection of systemic antioxidants based on SM has not been reported [15]. Crucially, using simple and portable equipment, PEC sensors can quantify the activity of antioxidants and the synergistic effect between different antioxidants in an intuitive and rapid way. As a result, PEC sensors are widely used in the analysis of food nutrients and the screening of active ingredients [16].

The excellent performance of PEC sensormostly depends on the properties of the photoactive material [17]. However, single semiconductors have high electron–hole recombination rates and low photocurrent signals as a result of limited visible-light absorption. To enhance photoelectric performance and overcome the limitations of semiconducting photosensitive materials, the development of multicomponent semiconductors and the design of multiple heterojunction structures have drawn attention [18,19]. In particular, the formation of a heterojunction can effectively improve the optical properties of semiconductors and promote electron–hole separation and transfer, thus expanding the light-absorbing window to the visible region and extending the operating life of the material [20].

Recently, nanorods having a large aspect ratio have attracted considerable attention because of their unique optical structure and electronic properties. Among the nanorod-forming materials, titanium dioxide (TiO_2_) exhibits a direct channel for electronic transmission and has a large specific surface area, making it especially suitable for various photocatalytic and sensing applications. However, TiO_2_ can only be excited by ultraviolet light, which comprises only 4–6% of the solar spectrum. Therefore, the modification of TiO_2_ to narrow the band gap is necessary to expand the absorption range to the visible region [21]. Copper sulfide (CuS) is a nontoxic semiconductor material that has a narrow band gap (1.2–2.0 eV) and high visible-light absorption coefficient, exhibiting particularly high absorption in the red and near-infrared regions [22]. Further, as a non-metallic semiconductor polymer, graphitic-phase carbon nitride (g-C_3_N_4_) has a large number of reactive sites and a unique band gap (2.7 eV). In addition, it is easily functionalized and doped with other semiconductors [23].

**Scheme 1:**
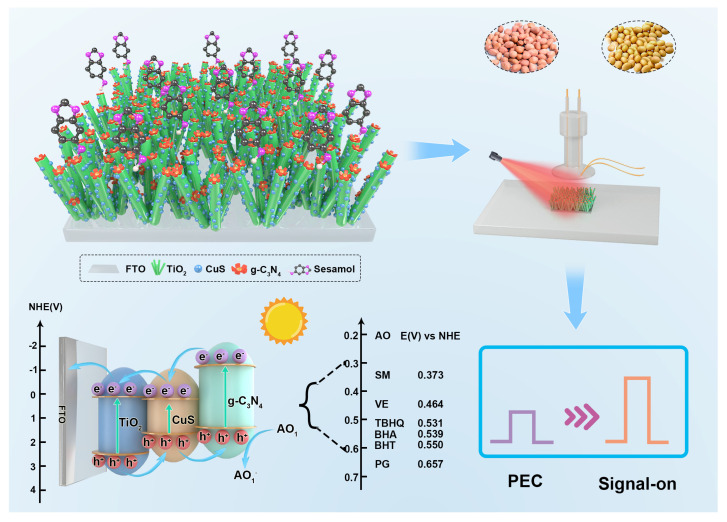
Schematic of antioxidant capacity analysis based on the g-C_3_N_4_/CuS/TiO_2_ PEC sensing platform. AO, antioxidant; FTO, fluorine-doped tin oxide; SM, sesamol; VE, vitamin E; TBHQ, *tert*-butyl hydroquinone; BHA, butyl hydroxyanisole; BHT, butylated hydroxytoluene; PG, propyl gallate.

To the best of our knowledge, this is the first study to construct a ternary heterojunction g-C_3_N_4_/CuS/TiO_2_ for the determination of SM. Briefly, a feasible and sensitive PEC sensing platform was constructed by depositing nanoparticles of CuS on TiO_2_ nanorods, followed by loading with g-C_3_N_4_ nanosheets that serve as the photoactive material. The sensing material was then applied for SM detection (Scheme 1). Further, the synergistic effects of SM and other antioxidants, including *tert*-butyl hydroquinone (TBHQ), vitamin E (VE), butyl hydroxyanisole (BHA), propyl gallate (PG), and butylated hydroxytoluene (BHT), were investigated and confirmed using the ternary heterojunction g-C_3_N_4_/CuS/TiO_2_ sensor. The findings of this study will aid the development of sensors for food safety, quality control, and food component compatibility testing and will aid the discovery of treatments for diseases caused by free radicals.

## 2. Experimental Procedure 

### 2.1. Synthesis of g-C_3_N_4_/CuS/TiO_2_ Composites 

TiO_2_ nanorods were synthesized using the classic hydrothermal method. Briefly, hydrochloric acid (15 mL), ultrapure water (15 mL), and titanium butoxide (500 μL) were added sequentially to a beaker and stirred for a few minutes. The mixture was transferred to a Teflon-lined autoclave containing a conductive fluorine-doped tin oxide (FTO) electrode. After react on at 150 °C for 5 h, the FTO electrode with TiO_2_ nanorods was rinsed with deionized water and annealed at 500 °C for 2 h at a heating rate of 2 °C min^−1^. Then, copper sulfate solution (0.25 µL, 0.1 mol L^−1^) and sodium thiosulfate solution (4.75 mL, 0.1 mol L^−1^) were added to a Teflon-lined autoclave containing an electrode modified with TiO_2_ nanorods and reacted at 120 °C for 5 h to obtain a sample, denoted CuS/TiO_2_. Using the same conditions, different doping amounts of CuS can be obtained by adding different proportions of copper sulfate and sodium thiosulfate solutions.

G-C_3_N_4_ was prepared by heating melamine at 550 °C for 2 h at a rate of 2 °C min^−1^. The products were washed three times with ultrapure water and ethanol sequentially, and dried at 60 °C. Then, g-C_3_N_4_ (10 mg) was added to ultrapure water (20 mL) and ultrasonicated for 3 h. The suspension was then centrifuged at a low speed for 10 min, and the supernatant was collected for further use.

To obtain the g-C_3_N_4_/CuS/TiO_2_ composite, the supernatant of g-C_3_N_4_ was added to a watch glass containing the CuS/TiO_2_ FTO electrode and soaked for 2 h at 70 °C. Then, the g-C_3_N_4_/CuS/TiO_2_ electrode was dried with nitrogen and calcined at 550 °C for 2 h under nitrogen atmosphere at a heating rate of 2 °C min^−1^. Under the same reaction conditions as those used to synthesize the composite materials, different loading amounts of g-C_3_N_4_ were obtained by adding different masses of g-C_3_N_4_ powder.

### 2.2. Antioxidant Capacity and Synergistic Effects

First, an antioxidant standard solution having a concentration of 10 mmol L^−1^ was prepared by dissolving SM, GA, CHA, EGC, EGCG, and PC in ethanol. For SM detection, the g-C_3_N_4_/CuS/TiO_2_ electrode was fastened to the PEC cell and suspended in an electrolyte comprising a phosphate-buffered saline (PBS) solution (3.0 mL; 0.1 mol L^−1^) of SM, and the photocurrent was measured at least three times at room temperature. The photocurrent was obtained using the formula: ∆*I* = *I_sample_* – *I_blank_*, where *I_sample_* is the photocurrent in the presence of the target and *I_blank_* is the photocurrent in the absence of the target. To investigate the synergistic effect of the SM-based antioxidant systems, 10 mmol L^−1^ mixtures of SM+VE, SM+BHA, SM+BHT, SM+TBHQ, SM+PG, SM+BHA+BHT+TBHQ+PG, and SM+BHA+BHT+TBHQ+PG+VE were prepared. The molar quantities of all antioxidants in these mixtures were equal. Similarly, VE, BHA, BHT, TBHQ, SM+VE, SM+BHA, SM+BHT, SM+TBHQ, SM+PG, SM+BHA+BHT+TBHQ+PG, and SM+BHA+BHT+TBHQ +PG+VE were added to the detection cell, and the photocurrent was measured. The synergistic effects can be calculated and quantified by comparing the photocurrents of a mixture of antioxidants.

## 3. Results and Discussion

### 3.1. Characterization of g-C_3_N_4_/CuS/TiO_2_ Composites

**Figure 1:**
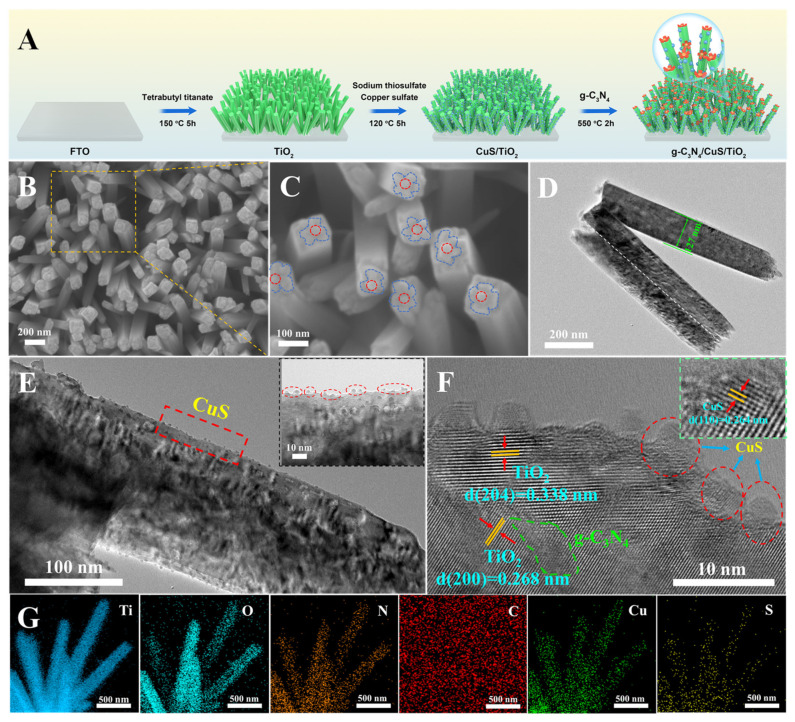
(**A**) Schematic showing the preparation of g-C_3_N_4_/CuS/TiO_2_. (**B**,**C**) Scanning electron microscopy (SEM) images, (**D**,**E**) transmission electron microscopy (TEM) images (inset: corresponding magnified picture of CuS nanoparticles), (**F**) high-resolution transmission electron microscopy (HR-TEM) images (inset: corresponding magnified HR-TEM image of CuS nanoparticles) and (**G**) energy-dispersive X-ray spectroscopy (EDX) of g-C_3_N_4_/CuS/TiO_2_.

Figure 1A shows a schematic of the g-C_3_N_4_/CuS/TiO_2_ composite synthesized in three steps. Scanning electron microscopy (SEM) images revealed that TiO_2_ grew uniformly on FTO and had a smooth surface (Figure S1A in Supplementary Materials). However, the surface became rough after the deposition of the CuS nanoparticles (Figure S1B). Figure 1B shows that the TiO_2_ nanorod structure did not change after loading with CuS and g-C_3_N_4_. As shown in Figure 1C, the tops of the nanorods have a flower-like appearance, which should increase the contact area available for reaction and is conducive to the collection and utilization of visible light. The transmission electron microscopy (TEM) image shown in Figure 1D was used to calculate the cross-sectional width of the g-C_3_N_4_/CuS/TiO_2_ composites: approximately 127 nm, whereas the length of the nanorods was approximately 500–900 nm. These dimensions should promote the longitudinal migration of photogenerated carriers. As shown in Figure 1E, the CuS nanoparticles were uniformly doped on the surfaces of the TiO_2_ nanorods, and the figure shows that the particle size ofCuS was less than 5 nm. Lattice fringes having interplanar spaces of 0.268, 0.338, and 0.264 nm can be clearly seen in Figure 1F, and these can be ascribed to the (200) and (204) crystal planes of anatase TiO_2_ and(110) crystal plane of CuS, respectively. Energy-dispersive X-ray spectroscopy (Figure 1G) measurements revealed the homogeneous distributions of Ti, O, Cu, S, C, and N in the g-C_3_N_4_/CuS/TiO_2_ composite, suggesting that the composite had been successfully synthesized.

**Figure 2:**
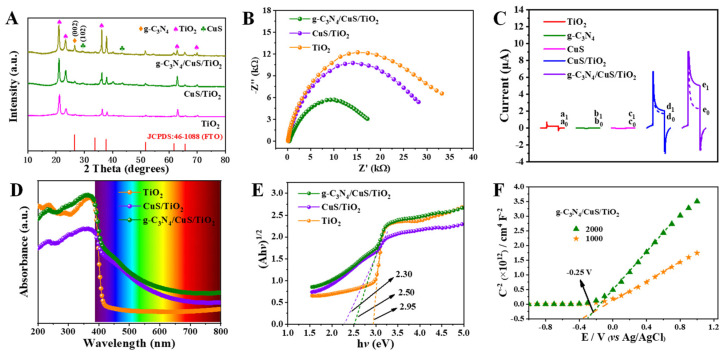
(**A**) X-ray powder diffractometry (XRD) and (**B**) Electrochemical impedance spectroscopy (EIS) plots of TiO_2_, CuS/TiO_2_, and g-C_3_N_4_/CuS/TiO_2_. (**C**) Photocurrent responses of TiO_2_, CuS, g-C_3_N_4_, CuS/TiO_2_, and g-C_3_N_4_/CuS/TiO_2_-modified FTO electrodes in the (a_0_–e_0_) absence and (a_1_–e_1_) presence of 123.46 μmol L^−1^ SM. (**D**) UV-Vis absorbance spectra and (**E**) Kubelka–Munk plots of TiO_2_, CuS/TiO_2_, and g-C_3_N_4_/CuS/TiO_2_. (**F**) Mott–Schottky plots of g-C_3_N_4_/CuS/TiO_2_ at frequencies of 1000 and 2000 Hz.

The crystallinity of g-C_3_N_4_/CuS/TiO_2_ was determined through X-ray diffraction (XRD) analysis (Figure 2A). The XRD pattern ofg-C_3_N_4_/CuS/TiO_2_ shows several characteristic peaks, corresponding to the (101), (103), (204), and (220) crystal planes of TiO_2_; (002) plane of g-C_3_N_4_; and (102) plane of CuS [24,25]. Furthermore, the XRD pattern of the as-prepared g-C_3_N_4_/CuS/TiO_2_ matches the patterns of standard hexagonal CuS (JCPDS No. 01-076-1725), anatase TiO_2_ (JCPDS No. 21-1272), and g-C_3_N_4_ (JCPDS No. 87-1526) well, suggesting that the stepwise synthesized composites were free of impurities. Next, X-ray photoelectron spectroscopy (XPS) measurements ofg-C_3_N_4_/CuS/TiO_2_ were conducted to analyze the electronic states of the surface elements. As shown in Figure S2, the high-resolution (HR)-XPS spectra of the elements ing-C_3_N_4_/CuS/TiO_2_ confirmed that the g-C_3_N_4_ thin-layer and CuS-nanoparticle-loaded TiO_2_ nanorod composite had been successfully synthesized via our designed route [26,27,28].

### 3.2. Photoelectrochemical Properties of the g-C_3_N_4_/CuS/TiO_2_ PEC Sensing Platform

Next, electrochemical impedance spectroscopy (EIS) was performed to determine the conductivities of the prepared samples. As shown in Figure 2B, g-C_3_N_4_/CuS/TiO_2_ yielded the smallest Nyquist semicircle radius, indicating that the composite had the lowest electron transfer resistance and higher conductivity than the other samples. This result can be attributed to the formation of a large number of heterointerfaces between the ternary semiconductor materials, which is conducive to charge transport.

The photocurrent response provides information about the optoelectronic properties and detection sensitivity of a material. Therefore, we investigated the photocurrents of the TiO_2_, g-C_3_N_4_, CuS, CuS/TiO_2_, and g-C_3_N_4_/CuS/TiO_2_-modified FTO electrodes (Figure 2C). Owing to the slow separation of photoinduced electron–hole pairs, the single semiconductor materials (i.e., TiO_2_, g-C_3_N_4_, and CuS) showed relatively low photocurrents both in the presence and absence of antioxidants. In contrast, the CuS/TiO_2_ electrode exhibited higher photoelectric activity: its photocurrent increased by 1.49 μA with respect to that of TiO_2_ alone, without the addition of the antioxidant. This is because CuS nanoparticles have high absorbance in the visible-light region. However, the photocurrent only increased by 0.32 μA after the addition of 123.46 μmol L^−1^ SM. Therefore, the sensitivity of the CuS/TiO_2_ electrode is low and is not sufficient to detect SM. Thus, further modification ofCuS/TiO_2_ by loading with g-C_3_N_4_ nanosheets is required. As expected, the photoelectric response and detection sensitivity of the ternary semiconductor composite (g-C_3_N_4_/CuS/TiO_2_) were significantly improved. Notably, the photocurrent response of the ternary semiconductor composite increased by 0.62 μA in relation to that of the binary semiconductor composite, and the photocurrent was increased by 2.68 μA in the presence of 123.46 μmol L^−1^ SM, suggesting that the ternary heterojunction PEC sensing platform is suitable for evaluating the antioxidant properties of SM. The significant increase in photocurrent can be attributed to two factors: (1) loading with g-C_3_N_4_ nanosheets increased the number of active sites for the reaction and enhanced light absorption, and (2) the heterojunctions in the ternary semiconductor composites promoted the reaction between antioxidants and holes and greatly improved the electron–hole separation. 

To determine the optical properties, UV-Vis absorbance spectroscopy was carried out, as shown in Figure 2D. TiO_2_ nanorods can only absorb light between 200 and 400 nm because of their wide band gap. However, the band gap was narrowed and more active sites for the reaction were produced after doping with CuS and g-C_3_N_4_, which increased the collection and utilization of light. Therefore, the ternary g-C_3_N_4_/CuS/TiO_2_ semiconductor exhibited greater light absorption at 200–800 nm, which is consistent with the EIS results. The ability of a sensor to detect antioxidants is strongly dependent on the band structure of the composites. Therefore, to achieve selective detection, the careful design of the valence band (VB) position of the semiconductor is required. As shown in Figure 2E, the relationship between the Kubelka–Munk function and the photon energy of the samples can be obtained from the UV-Vis absorbance spectra. The VB values ofTiO_2_, CuS/TiO_2_, and g-C_3_N_4_/CuS/TiO_2_ are 2.95, 2.30, and 2.50 eV, respectively. The Mott–Schottky (M-S) plots ofg-C_3_N_4_/CuS/TiO_2_ semiconductor exhibited positive slopes (Figure 2F), suggesting that the composites are n-type heterostructures, and the flat-band potential (V*_fb_*) was −0.25 V (vs. Ag/AgCl). In addition, the conduction band (CB) of the n-type semiconductor is 0.1 V smaller than its V*_fb_*; thus, the CB of g-C_3_N_4_/CuS/TiO_2_ is −0.35 V (vs. Ag/AgCl).

### 3.3. Optimization of Experimental Conditions

The intrinsic properties of photosensitive materials are the main factors affecting the photocurrent signal, but other factors, such as the degree of doping of the semiconductor material, laser wavelength, and applied voltage, cannot be ignored. Optimization experiments were performed inPBS solution containing 123.46 μmol L^−1^ SM. As shown in Figure S3A, we investigated the influence of the amount of deposited CuS on the photocurrent. Crucially, an appropriate amount of CuS nanoparticles will increase the photocurrent, but an excessive amount could result in a drastic decrease in photocurrent. Based on the experiments, a doping level of 5% was selected to prepare the CuS/TiO_2_ composite. The mass ofg-C_3_N_4_ is another key factor that affects the properties of semiconductors. As shown in Figure S3B, g-C_3_N_4_ nanosheets provide more active sites for the reaction, and those prepared using a mass of 10 mg generated the highest photocurrent. As shown by the UV-Vis spectra, the g-C_3_N_4_/CuS/TiO_2_ exhibited different absorbances in different bands. In particular, as an excitation light source, 630 nm red light has strong penetration. In addition, red light accounts for a large proportion of sunlight, suggesting its suitability for enhancing the sensitivity of the sensor for SM detection (Figure S3C). As shown in Figure S3D, there is a significant gradient between the semiconductor and FTO electrode in the initial state. When a potential is applied, electrons flow rapidly from the conductor band of the semiconductor to the FTO electrode to generate a photocurrent signal. The current at 0 V was 77.3% of that at +0.08 V, indicating that the PEC sensor has adequate sensitivity for SM detection. Therefore, 0 V was chosen as the working voltage for further experiments.

### 3.4. Antioxidant Assay and Detection Mechanism of the g-C_3_N_4_/CuS/TiO_2_ PEC Sensing Platform

**Figure 3:**
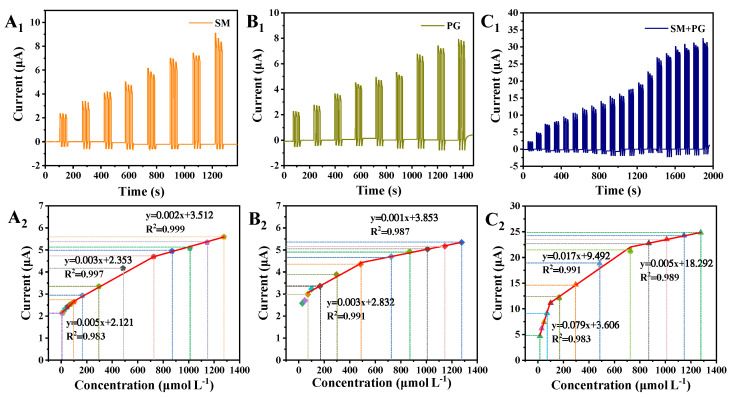
Photocurrent response curves of SM (**A_1_**), PG (**B_1_**), and SM+PG in equal proportions (**C_1_**) generated for the g-C_3_N_4_/CuS/TiO_2_-based PEC sensing platform at different analyte concentrations. Calibration plot of photocurrent versus different concentrations of SM (**A_2_**), PG (**B_2_**), and SM+PG (**C_2_**).

To analyze the detection performance of the as-prepared g-C_3_N_4_/CuS/TiO_2_ PEC sensor upon 630 nm laser irradiation, the photocurrent signal was measured in PBS solutions (0.1 mol L^−1^) having different concentrations of SM, PG, and the SM+PG mixture under the optimized assay conditions. As shown in Figure 3A_2_, the photoresponse in SM detection exhibited a linear relationship with a detection limit (S/N = 3) of 1.8 μmol L^−1^, with three linear regression equations obtained for three concentration ranges, as follows: *y* = 0.005*x* + 2.121, *R*^2^ = 0.983 (2.157–100.196 μmol L^−1^), *y* = 0.003*x* + 2.353, *R*^2^ = 0.997 (100.196–725.196 μmol L^−1^), and *y* = 0.002*x* + 3.512, *R*^2^ = 0.999 (725.196–1276.667 μmol L^−1^). Compared with the SM detection performance of previously reported methods (Table S2), the SM detection performance of the g-C_3_N_4_/CuS/TiO_2_ PEC sensing platform is excellent. In particular, the proposed sensing platform exhibits a distinctly higher detection sensitivity and a wider linear detection range, which completely satisfy actual sample detection requirements. The photocurrent response of the g-C_3_N_4_/CuS/TiO_2_ PEC sensor to PG was lower than that of the SM sensor (Figure 3B_1_), and two linear relationships were obtained (Figure 3B_2_): *y* = 0.003*x* + 2.832, *R*^2^ = 0.991 (71.787–484.812 μmol L^−1^) and *y* = 0.001*x* + 3.853, *R*^2^ = 0.987 (484.812–1276.667 μmol L^−1^). Concerning the antioxidant mixtures of SM+PG, the photoresponse displayed favorable linear relationships in the three concentration ranges (Figure 3C_1_), and the corresponding linear equations are *y* = 0.079*x* + 3.606, *R*^2^ = 0.983 (15.990–94.627 μmol L^−1^); *y* = 0.017*x* + 9.492, *R*^2^ = 0.991 (94.627–735.132 μmol L^−1^); and *y* = 0.005*x* + 18.292, *R*^2^ = 0.989 (735.132–1276.667 μmol L^−1^), respectively. These results indicate that the photoelectric response from the two-component mixture of antioxidants was significantly greater than the combined photocurrents of individual components at the same concentration. This indicates a synergistic effect between SM and PG, especially at high concentrations. Based on these findings, we investigated the synergistic effects of SM and VE, BHA, BHT, and TBHQ. As shown in Figure S4, SM was mixed in pairs with these four typical antioxidants, and the obtained trends are similar to that of the two-component mixtures of SM+PG. Presumably, an additive effect occurs at low concentrations, and a synergistic effect occurs at high concentrations. It has been reported that long distances suppress electron transport between antioxidants at low concentrations. Therefore, at relatively high concentrations, frequent electron transfer and the coupling with the oxidant may promote the regeneration of antioxidants, thus resulting in a more pronounced synergistic effect [29,30,31].

**Figure 4:**
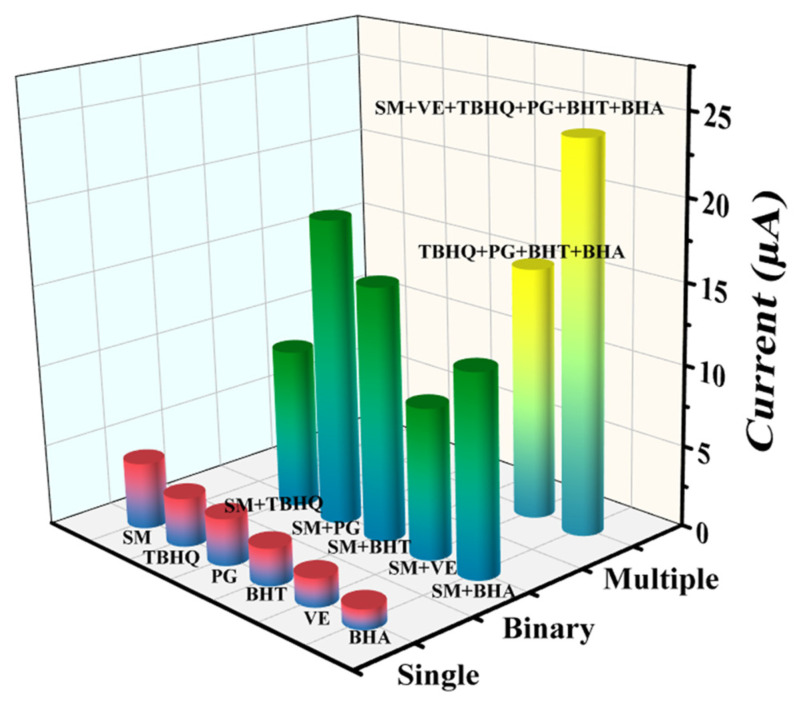
Photocurrents of the g-C_3_N_4_/CuS/TiO_2_ PEC sensor in the presence of 484.812 μmol L^−1^ SM, VE, TBHQ, BHA, BHT, PG, and a mixture of SM and other antioxidants in equal molar ratios.

To understand the sensing principle of our sensor, as well as the observed synergistic effects, the electrochemical properties and photoresponses of the antioxidants were investigated in detail. As shown in Table S1, cyclic voltammetry of the antioxidants at a concentration of 484.812 μmol L^−1^ was carried out, yielding the following redox potentials: SM (0.373 V) < VE (0.464 V) < TBHQ (0.531 V) < BHA (0.539 V) < BHT (0.550 V) < PG (0.657 V). SM exhibited the smallest redox potential, suggesting its ability to reduce antioxidants having higher redox potentials [29,32]. As shown in Figure 4 and Table S1, the synergistic effect of binary antioxidant mixtures SM+VE, SM+TBHQ, SM+BHA, SM+BHT, and SM+PG resulted in increases of 1.57-, 1.38-, 2.31-, 2.42-, and 2.67-times, respectively, compared to the sum of the single antioxidant. This result is similar to the trend in the redox potential of the antioxidants. However, the synergistic effect of SM+VE was more obvious than that of SM+TBHQ, which is inconsistent with the increasing trend in redox potential. This is because both SM and VE are natural antioxidants, and their molecular structure and electronic properties (nucleophilic, electrophilic, ionization potential, and phenolic bond dissociation energy) are similar, thereby showing better performance [33]. Furthermore, we investigated the antioxidant effects of mixtures of natural antioxidants (SM+VE), four artificial antioxidants (BHA+BHT+TBHQ+PG), and six natural and artificial antioxidants (SM+VE+BHA+BHT+TBHQ+PG). All the samples showed improvements in the photocurrent responses, SM+VE, BHA+BHT+TBHQ+PG, and SM+VE+BHA+BHT+TBHQ+ PG showed increases of 1.57-, 1.64-, and 1.56-times, respectively, in relation to the sum of photoresponses of all single antioxidants. These results indicate that there are synergistic and antagonistic effects between the antioxidant molecules. In particular, there is a negative correlation between complexity of the antioxidant components and the antioxidant effect; thus, it is crucial to select a suitable formula to maximize the antioxidant performance [34]. These data also indicate that natural antioxidants exhibit good antioxidant properties and could be used to replace synthetic antioxidants as food stabilizers.

Based on the above results, we conclude that coupling oxidation plays a key role in the synergism of the studied antioxidants: specifically, it reduces the potential difference between pairs of antioxidants and promotes their regeneration, thus improving the performance of binary mixtures of antioxidants. Additionally, the heterojunctions in the g-C_3_N_4_/CuS/TiO_2_ composite promote the reaction between antioxidants and holes, thus improving the separation and transition of electrons and holes (Scheme 1) and amplifying the detectable signal. Therefore, the g-C_3_N_4_/CuS/TiO_2_-modified platform could be applied for the detection and evaluation of the synergistic effect of SM based antioxidants.

### 3.5. Detection Selectivity Stability and Reusability ofg-C_3_N_4_/CuS/TiO_2_ PEC Sensing Platform

**Figure 5:**
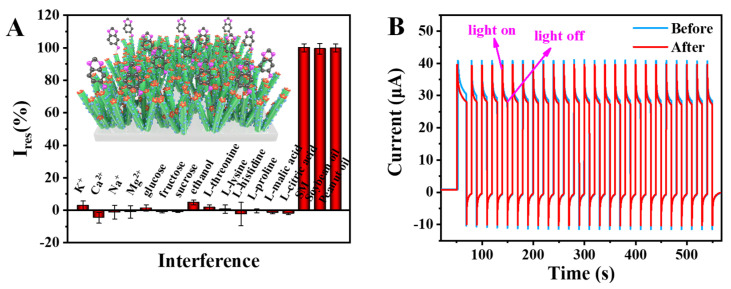
(**A**) Samples and interferences test in the presence of fructose, glucose, sucrose, l-malic acid, l-citric acid, ethanol, l-threonine, l-proline, l-lysine, and l-histidine, as well as Na^+^, K^+^, Mg^2+^, and Ca^2+^, on the photocurrent response of the g-C_3_N_4_/CuS/TiO_2_-based PEC sensor. (**B**) Detection stability and reusability (after 15 days) of the g-C_3_N_4_/CuS/TiO_2_-modified FTO electrode in the presence of 1270.574 μmol L^−1^ SM. The PEC sensor was operated at 0 V under 630 nm light excitation in 0.1 mol L^−1^ PBS (pH = 7.4).

Sensor selectivity depends largely on the band structure of the composite. Specifically, an appropriate VB can oxidize target species with a lower redox potential, but does not react with the sugars and amino acids in food. As shown in Figure 5A, we tested the effect of 18 times of fructose, glucose, sucrose, L-malic acid, L-citric acid, ethanol, L-threonine, L-proline, L-lysine, and L-histidine, as well as 700 times of Na^+^, K^+^, Mg^2+^ and Ca^2+^ on photocurrent based on g-C_3_N_4_/CuS/TiO_2_ PEC sensor with 123.46 μmol L^−1^ SM. No detectable signals (<10%) corresponding to the interfering agents were detected, suggesting that the PEC sensor hashigh SM detection selectivity. Furthermore, we had tested the photocurrent of real samples (soybean oil and peanut oil) in the same conditions of SM, there are insignificant change in the photocurrent response, which further verified the feasibility of the sensor in the analysis of antioxidant capacity of food.

Sensor stability and reusability are also important criterions to evaluate the performance ofPEC sensor, especially for practical, long-term detection in real samples. As shown in Figure 5B, the g-C_3_N_4_/CuS/TiO_2_ PEC sensor was monitored using amperometry with 500 s of “on–off–on” cycling in the presence of 1270.574 μmol L^−1^ SM, which showed no obvious decrease in the photoelectric signal. After 15 days, the photocurrent signal was reduced to 96.8% of the initial state, indicating that the sensor has excellent stability and reusability.

### 3.6. SM Detection in Soybean and Peanut Oils

Oils and fats naturally become rancid as a result of oxidation during storage or processing. The addition of synthetic or natural antioxidants can slow down lipid peroxidation and speed up the scavenging of free radicals [11,35,36]. In view of the high selectivity of the g-C_3_N_4_/CuS/TiO_2_ PEC sensor platform, the applicability of the sensor for use with real oil samples was investigated using soybean and peanut oils containing various SM concentrations. The corresponding calibration curve is shown in Figure 3A. As listed in Table 1, both soybean and peanut oil samples exhibited high recoveries and low relative standard deviations (RSD; <3%) at different SM concentrations, indicating excellent detection precision and accuracy. Therefore, the natural antioxidant SM showed excellent antioxidant properties and could replace synthetic antioxidants as quality stabilizers for edible oils, which is important for food safety, quality control, and ingredient screening.

## 4. Conclusions

We developed a novel red-light-mediated signal-on PEC sensor based on a g-C_3_N_4_/CuS/TiO_2_ ternary heterojunction for a highly sensitive analysis of the synergistic antioxidant effects of SM and other antioxidants. The g-C_3_N_4_/CuS/TiO_2_ heterojunction forms a Schottky barrier that facilitates electron transfer. When SM and other antioxidants are present, they scavenge the holes in the VB of g-C_3_N_4_/CuS/TiO_2_, suppressing the recombination of photogenerated charge carriers, thus increasing the PEC photocurrent. The sensor also allowed the rapid evaluation of the synergistic effects of SM and VE, BHA, BHT, TBHQ, and PG. The synergistic antioxidant reaction of multiple antioxidants is mainly driven by coupling oxidation, which enhances the total antioxidant performance by lowering the potential difference between the antioxidants. We found that SM reduces the free radicals of other antioxidants and promotes their regeneration because of its low redox potential. Overall, the g-C_3_N_4_/CuS/TiO_2_ PEC sensor exhibited high sensitivity, good selectivity, and stability, and was successfully applied to the detection of SM in soybean and peanut oils. The reported sensor has applications in nutrition analysis and the development of nutritional additives, which could help improve the quality of life of consumers. In addition, the sensor has potential uses in food fraud analysis, as well as medical research.

## Figures and Tables

**Table 1:** SM Concentrations in Soybean and Peanut Oil Samples Detected Using the g-C_3_N_4_/CuS/TiO_2_-based PEC Sensor.

Sample	SM Added (μM)	SM Found (μM)	RSD (%)	Recovery (%)
Soybean oil	4	3.93 ± 0.01	0.31	98.28
	20	20.40 ± 0.20	0.98	102.00
Peanut oil	4	3.97 ± 0.09	2.30	99.16
	20	20.40 ± 0.53	2.59	102.00

## Data Availability

Not applicable.

## Supplementary Materials

The following supporting information can be downloaded at: https://www.mdpi.com/article/10.3390/bios13090859/s1: Reagents; Apparatus; Figure S1: SEM images of TiO_2_ and CuS/TiO_2_; Figure S2: XP spectra of g-C_3_N_4_/CuS/TiO_2_; Figure S3: Optimization of experimental conditions; Figure S4: Photocurrent response curves; Table S1: Electrochemical characteristics and synergistic effects; Table S2: Comparison of the SM detection performance of various methods. References [10,37,38,39] are cited in Supplementary Materials.
