# Supplementary material for: Ternary Heterojunction Graphitic Carbon Nitride/Cupric Sulfide/Titanium Dioxide Photoelectrochemical Sensor for Sesamol Quantification and Antioxidant Synergism
Source: Biosensors (Basel). 2023 Aug 30;13(9):859. doi: 10.3390/bios13090859 (PMC10526488; doi:10.3390/bios13090859)
Supplement: Supplementary file 1 [file biosensors-13-00859-s001.zip › biosensors-2578281-supplementary.pdf]

Supporting Information

Ternary Heterojunction g-C_3N_4/CuS/TiO_2 Photoelectrochemical Sensor for Sesamol Quantification and Antioxidant Synergism

Likun Huang [1], Jingshi Yang [1], Zhishan Liang [1], Ruilian Liang [1], Hui Luo [1], Zhonghui Sun [1], Dongxue Han*[1,2], Li Niu[1,3]

[1] Guangzhou Key Laboratory of Sensing Materials & Devices, *Center for Advanced Analytical Science, School of Chemistry and Chemical Engineering, Guangzhou University, Guangzhou 510006, China.*

[2] *Guangzhou Provincial Key Laboratory of Psychoactive Substance Monitoring and Safety, Anti-Drug Technology Center of Guangdong Province, Guangzhou 510230, China.*

[3] *School of Chemical Engineering and Technology, Sun Yat-sen University, Zhuhai 519082, China*

** Corresponding authors: Dongxue Han (E-mail: dxhan@gzhu.edu.cn)*

This material includes:

Experimental section	Reagents; Apparatus.
Figure S1	SEM images of TiO_2 and CuS/TiO_2.
Figure S2	XPS of g-C_3N_4/CuS/TiO_2.
Figure S3	Optimization of Experimental Conditions.
Figure S4	Photocurrent response curves.
Table S1	Electrochemical characteristics and synergistic effects.
Table S2	Comparison of the SM detection performance of various methods.

Experimental section

Reagents

Titanium butoxide (98.0%), copper sulfate pentahydrate (99.0%), sodium thiosulfate pentahydrate (99.0%), melamine, disodium hydrogen phosphate and sodium dihydrogen phosphate were purchased from Shanghai Macklin Biochemical Co., Ltd. Hydrochloric acid, potassium ferricyanide, acetone, fructose, glucose, sucrose, L-malic acid, L-citric acid, ethanol, L-threonine, L-proline, L-lysine and L-histidine were purchased from Aladdin Reagent Database, Inc. (Shanghai, China). Sesamol, Tert-butyl hydroquinone (TBHQ), Vitamin E (VE), Butyl hydroxyanisole (BHA), Propyl gallate (PG) and Butylated hydroxytoluene (BHT) were obtained from Sigma–Aldrich. Fluorine-doped tin oxide (FTO) glass was purchased from Jinge Co., Ltd. (Wuhan, China).

Apparatus

The sample morphologies were characterized using field-emission scanning electron microscopy (FE-SEM; JSM-7001F) and (high-resolution) transmission electron microscopy [(HR)-TEM; JEOL JEM-2100F] operating at 200-kV acceleration. The sample crystallinity was measured using powder X-ray diffraction (XRD; PW3040/60 diffractometer). The surface electronic states were analyzed using X-ray photoelectron spectroscopy (XPS; Thermo ESCALAB 250Xi), and all the binding energies were referenced to the C 1*s* peak at 284.8 eV. The ultraviolet–visible (3600 plus spectrometer; Shimadzu) absorption spectra were recorded using $BaSO_4$ as a reference. The sample electrical resistances were measured using electrochemical impedance spectroscopy (EIS; 1255 B frequency response analyzer; Solartron Inc., U.K.) at an amplitude of 5 mV from 10^{-1} to 10^5 Hz in a mixed electrolyte solution comprising $[Fe(CN)_6]^{3-/4-}$ (1 mmol L^{-1}) and KCl (0.1 mol L^{-1}). All the PEC measurements were performed using an electrochemical workstation (CHI 660E; Shanghai Chenhua; China) and a standard

three-electrode system comprising a modified FTO working electrode, a commercial Ag/AgCl reference electrode, and a platinum wire counter electrode. FTO slices were cleaned by immersion in an aqueous NaOH solution (2.0 mol L^{-1}). For the PEC analysis, PBS (0.1 mol L^{-1}, PH=7.4) was used as a supporting electrolyte. The electrode was fastened to the PEC cell (containing a certain SM concentration) equipped with a 630-nm light irradiation, exhibiting a working potential of 0 V. The photocurrent was measured at least three times and all the experiments were performed at room temperature.

Results and discussion

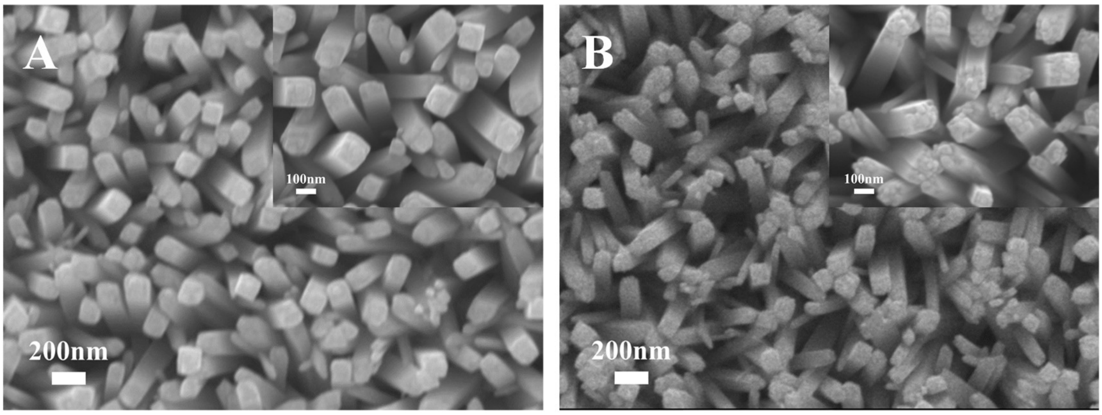

Figure S1. SEM images of TiO_2 (A) and CuS/TiO_2 (B).

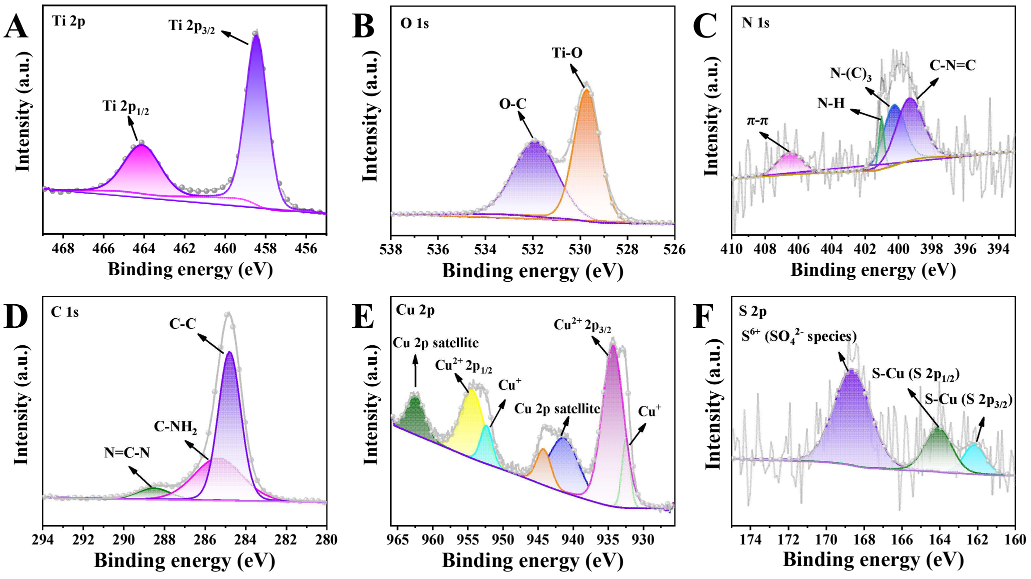

Figure S2. X-ray photoelectron spectroscopy (XPS) of g-C_3N_4/CuS/TiO_2. High-resolution spectra in (A) Ti 2p, (B) O 1s, (C) N 1s, (D) C 1s, (E) Cu 2p, and (F) S 2p.

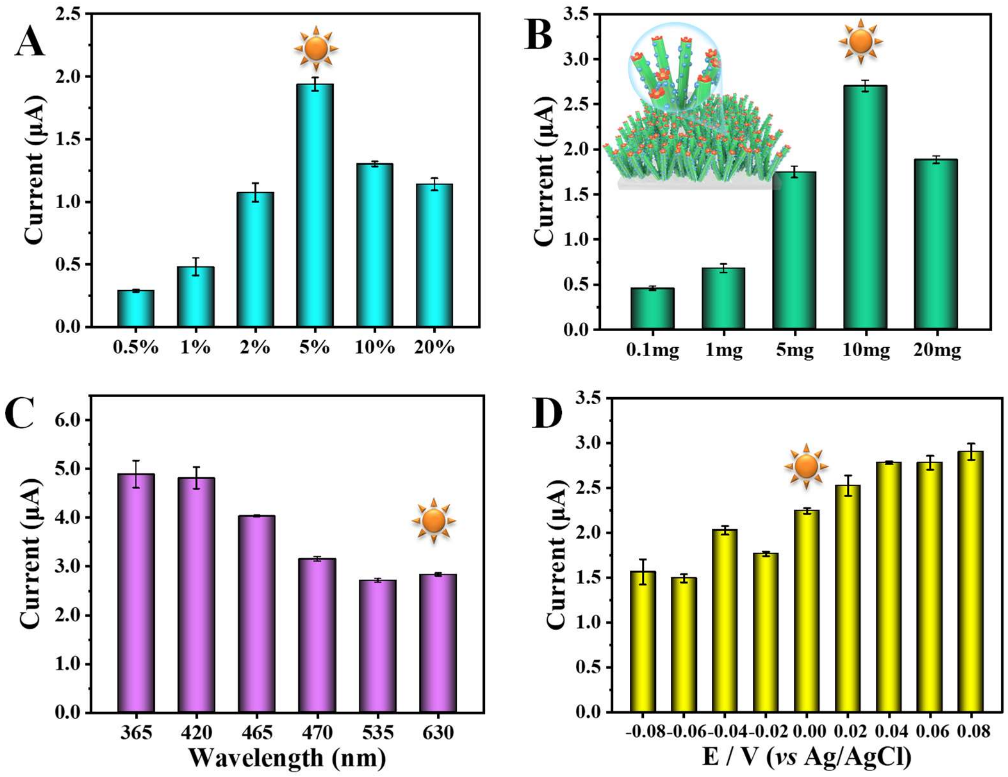

Figure S3. (A) The effect of different CuS deposition amount on photocurrent of g-C_3N_4/CuS/TiO_2 FTO electrode. (B) Photocurrent responses of a series of g-C_3N_4 doped g-C_3N_4/CuS/TiO_2-modified FTO electrode. (C) Photocurrent responses of g-C_3N_4/CuS/TiO_2-modified FTO electrode under different function of wavelength and (D) Influence of applied potential on g-C_3N_4/CuS/TiO_2-modified FTO electrode photocurrent response. All condition optimization experiments were tested in 0.1 mol L^{-1} PBS buffer solution containing 123.46 μmol L^{-1} SM.

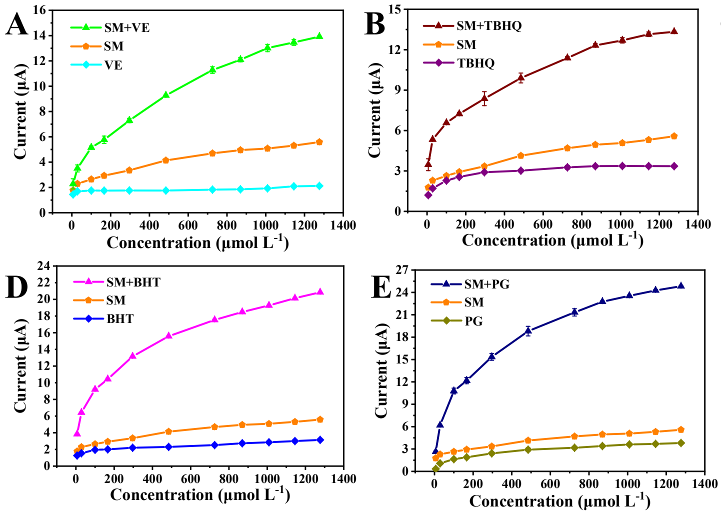

Figure S4. Photocurrent response curves generated from g-C_3N_4/CuS/TiO_2-based PEC sensing platform of single and binary mixed antioxidants in equal proportions at different concentrations: (A) SM, VE, and SM+VE. (B) SM, TBHQ, and SM+TBHQ. (C) SM, BHA, and SM+BHA. (D) SM, BHT, and SM+ BHT. (E) SM, PG, and SM+PG.

Table S1. The electrochemical properties and synergistic effects of g-C_3N_4/CuS/TiO_2 PEC sensor in the presence of 484.812 μmol L^{-1} SM, VE, TBHQ, BHA, BHT, PG and a mixture of SM and other antioxidants with the same mole rate (1:1).

Antioxidant	SM	VE	TBHQ	BHA	BHT	PG
**Molecular structure**	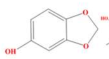	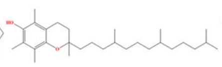	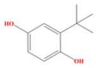	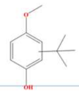	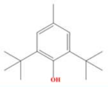	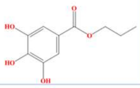
**Solubility**	**oil-soluble**	**oil-soluble**	**oil-soluble**	**oil-soluble**	**oil-soluble**	**oil-soluble**
**Cyclic voltammetry**	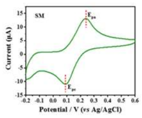	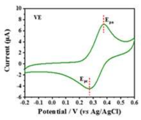	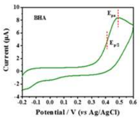	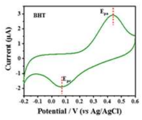	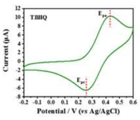	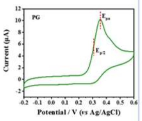
**E_{pa} vs NHE (V)**	**0.448**	**0.648**	**0.583**	**0.563**	**0.636**	**0.696**
**$E_{p/2}$ (V)**				**0.307**		**0.411**
**E_{pc}(V)**	**0.090**	**0.072**	**0.271**		**0.256**	
**(E_{pa}+$E_{p/2}$)/2 *vs* Ag/AgCl (V)**				**0.332**		**0.45**
**(E_{pa}+E_{pc})/2 *vs* Ag/AgCl (V)**	**0.166**	**0.257**	**0.324**		**0.343**	
**Redox potential vs. NHE (V)**	**0.373**	**0.464**	**0.531**	**0.539**	**0.550**	**0.657**
**Synergism (484.812 μmol L^{-1})**	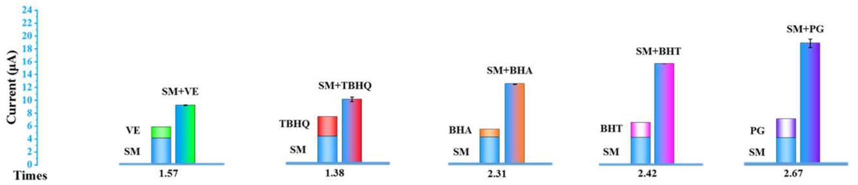 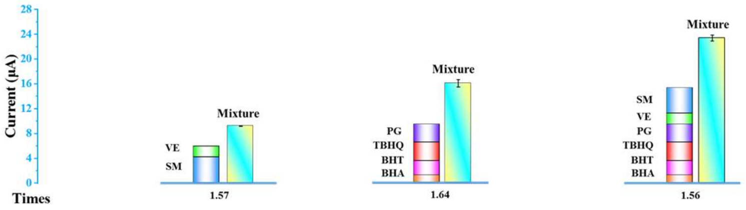					

Table S2. Comparison of the SM detection performance of various methods.

Method	Linear range	LOD	Reference
Electrochemical detection	1.7-67.0 μM	--	[1]
Electroanalytical assay	3.0-140.0 μM	0.71 μM	[2]
High Performance Liquid Chromatography	5.0-500.0 mg kg^{-1}	0.02 mg kg^{-1}	[3]
Spectrofluorometer	2.4-1200.0 μM	7.8 μM	[4]
PEC sensor	2.0-1277.0 μM	1.8 μM	This work

References:

[1]. Ohno, K.-i.; Sato, K.; Kumano, M.; Watanabe, K.; Fujimura, T. Electrochemical detection of sesamol dimer and its application to measurement of radicals. *Anal. Sci.* **2021**, *37* (4), 633-635.

[2]. Morawska, K.; Festinger, N.; Chwatko, G.; Głowacki, R.; Ciesielski, W.; Smarzewska, S. Rapid electroanalytical procedure for sesamol determination in real samples. *Food Chem.* **2020**, *309*, 125789.

[3]. Liu, W.; Zhang, K. D.; Qin, Y. Q.; Yu, J. J. A simple and green ultrasonic-assisted liquid–liquid microextraction technique based on deep eutectic solvents for the HPLC analysis of sesamol in sesame oils. *Anal. Method.* **2017**, *9*, 4184.

[4]. Liu, H. L.; Wu, D.; Liu, Y. L.; Zhang, H. J.; Ma, T. Z.; Aidaerhan, A.; Wang, J.; Sun, B. G. Application of an optosensing chip based on molecularly imprinted polymer coated quantum dots for the highly selective and sensitive determination of sesamol in sesame oils. *J. Agric. Food Chem*. **2015**, *63*, 2545-2549.
